# Genomic insights into habitat adaptation of *Lactobacillus* species

**DOI:** 10.1007/s11274-025-04275-0

**Published:** 2025-02-04

**Authors:** Alejandra Mejía-Caballero, Rafael López-Sánchez, Blanca Ramos-Cerrillo, Alejandro Garciarrubio, Lorenzo Segovia

**Affiliations:** Departamento de Ingeniería Celular y Biocatálisis, Instituto de Biotecnología, Universidad Nacional Autónoma de México, Cuernavaca, Morelos México

**Keywords:** Lactobacillus, Comparative genomics, Pangenomics, Habitat Adaptation, Domestication

## Abstract

*Lactobacillus* is one of the most important genera within the lactic acid bacteria group, due to its importance in the food industry and the health field. This diversity can be explained either by their radiation in different environments or by the domestication process in artificial habitats, such as fermented foods. In this study, we performed a comparative genomic analysis of 1020 *Lactobacillus* genomes, categorizing them into five broad habitats: insects, vertebrates (including humans and animals), vegetables, free-living environments, and dairy products. Utilizing phylogenetic relationships, genomic distances, and gene presence/absence profiles, we identified distinct clustering patterns associated with specific environmental adaptations. Notably, species within the *Lactobacillus delbrueckii* clade exhibited GC content variations fivefold greater than those observed in other bacterial genera, indicating significant genomic divergence. Insect-associated species showed a strong correlation between genes for carbohydrate utilization and those for amino acid biosynthesis across all habitats. However, individual gene analyses revealed no consistent correlation between habitat adaptation and phylogenetic proximity, suggesting that *Lactobacillus* employs strain-specific adaptive mechanisms rather than universal genetic markers. Notably, around 50% of the genes associated with specific habitats are hypothetical. Our findings highlight the genomic complexity of *Lactobacillus*, driven by diverse adaptive strategies, and underscore the need for more comprehensive sampling to fully elucidate the evolutionary dynamics within this important genus.

**Supplementary Information:**

The online version contains supplementary material available at 10.1007/s11274-025-04275-0.

## Introduction

Lactic acid bacteria (LAB) are a heterogeneous group of Gram-positive bacteria located phylogenetically in the Clostridia branch (De Filippis et al. 2020), where lactic acid is the main metabolic end-product of sugar fermentation. LAB plays a key role in the global food supply, performing the main bioconversions in fermented dairy products, meats, and vegetables (Makarova and Koonin 2007). They are also members of the gut microbiome of humans, animals, and insects, but their origin, role, and potential activities are still widely discussed (De Filippis et al. 2020).

*Lactobacillus* is a genus of significant importance to human and animal health, as well as food production, particularly for its roles in fermented foods and probiotic properties. Species within this genus thrive in diverse environments and are key members of the microbiota in animals and insects. In humans, certain species exhibit remarkable specialization, such as *Lactobacillus iners*, while others, like *L. delbrueckii* and *L. helveticus*, are renowned for their roles in dairy fermentation. The broad environmental distribution of *Lactobacillus* makes it an excellent model for studying genome modifications. These adaptations offer valuable insights into evolutionary processes, including speciation and habitat-specific adaptations driven by domestication.

Different works have tried to explain this diversity and have attributed it to adaptation to the wide range of niches that these species can inhabit (O’Sullivan et al. 2009; Zheng et al. 2015). They can be found in environments with a high concentration of proteins, such as dairy or meat fermentations (Broadbent et al. 2012; Nyquist et al. 2011), associated with vegetables where the main nutrients are complex carbohydrates (Wuyts et al. 2019), and also with vertebrates or insects (Kainulainen et al. 2015; Neveling et al. 2012; Vuong and McFrederick 2019). The great genetic diversity of the genus reflects all the different environments in which they can survive and proliferate. These adaptations are also reflected in a decrease in genome size, accompanied by several specific events of horizontal gene transfer (HGT) that allow them to adapt to certain niches (Albalat and Cañestro 2016; Makarova and Koonin 2007; Petrova et al. 2017). For example, LABs adapted to grow in dairy products have a higher number of proteases and peptidases (Griffiths and Tellez 2013). In comparison, those associated with vegetables have a higher number of genes associated with the consumption of carbohydrates (Vrancken et al. 2008), giving them the advantage of growing better in each environment.

Although adaptation processes to these niches may explain part of the diversity, they do not fully describe the scope seen in lactobacilli, as there are discrepancies in terms of genome size, GC content, and genetic diversity (Griffiths and Tellez 2013). An alternative explanation is that this diversity comes from evolutionary changes, meaning that these differences are related to taxonomic diversity, rather than adaptation; speciation events are unlikely since fermented foods are, by definition, recent artificial environments. For this reason, four general niches have been reported for the lactobacilli group, excluding artificial environments: (1) vertebrate-adapted, (2) insect-adapted (3) free-living, and (4) nomadic (Duar et al. 2017).

The taxonomy of *Lactobacillus* has been extensively revised in recent years and there have been many proposals for its reclassification (Claesson et al. 2008; Salvetti et al. 2012). Recently, Zheng et al. (2020), evaluated the taxonomy considering the core genome phylogeny, pairwise average amino acid identity, clade-specific signature genes, physiological criteria, and the ecology of the genus. Based on these parameters, they propose the reclassification of the genus into 23 new genera, conserving the *Lactobacillus* genus with the type species *Lactobacillus delbrueckii*, and merging the two closest families *Leuconostocaceae* and *Lactobacillaceae* (Zheng et al. 2020).

We suggest that although fermented foods are defined as artificial environments (Duar et al. 2017), *Lactobacillus* species have acquired certain functions that allow them to proliferate in these habitats. In this sense, we propose a specific habitat classification that could help to have a strain-level resolution in identifying habitat-related functions. We found that some of these functions seem to originate mainly from horizontal transfer events (HGTs) of species that are not necessarily linked to that habitat.

Because *Lactobacillus* is so closely involved in the production of fermented foods, they live in constantly changing environments and undergo genomic rearrangements. Therefore, it is necessary to understand which characteristics of the genome are related to evolutionary events and which are involved in recent adaptations to artificial niches. Understanding this complex relationship will allow us to better understand the physiology of this genus and improve its use in industrial and health applications.

## Methods

### Data collection

For the phylogenetic analysis of the *Lactobacillus* genus, 45 reference genomes were downloaded from the NCBI database (Table S1). For the pangenomic and gene-specific analyses, 1020 complete genomes, including those reported as uncultured and sp., were also downloaded from NCBI (Table S2). We analyzed all the genomes together and also divided by species using PyANI (Pritchard et al. 2019) to evaluate the taxonomic coherence and identify potential misclassification. Clearly *Lactobacillus* sp. and the uncultured lactobacilli are not consistent, as expected as they are defined by exclusion. *L. intestinalis* has an overall similarity of 92%, *L. jensenii* has two unrelated clusters (one having only two strains), and *L. johnsonii* is the most heterogenous with all strains having a higher than 90% ANI among themselves. All lactobacilli have a high ANI value (> 89%), showing a good level of coherence. The CheckM (Parks et al. 2015) analysis of all strains showed a range of 100–70% of completeness, the latter belonging exclusively to MAGs. All genomes exhibited less than 10% contamination. We kept all genomes, even substandard ones, as they comprised the broadest collection of species encompassing different habitats.

### Phylogenetic analysis

We used amino acid sequences and the Phylophlan 3.0 pipeline to calculate trees (Asnicar et al. 2020) for the phylogeny analysis of the *Lactobacillus* genus and *L. delbrueckii* species. We used the Phylophlan database (Segata et al. 2013) which includes 400 universal marker genes, and Diamond v0.9.24.125 (Buchfink et al. 2021) to map the database with our proteomes. Multiple sequence alignments (MSA) were performed with MUSCLE v3.8.31 (Edgar 2004) and trimAI software v1.4.rev22 (Capella‑Gutiérrez et al. 2009) for trimming of gappy regions. Finally, we used the Maximum Likelihood estimation with the software IQ-TREE v2.0.6 (Nguyen et al. 2015) and RaxML v.8 (Stamatakis 2006), for the calculation of the trees.

We used *Lactiplantibacillus pentosus* ZFM94 and *Lactiplantibacillus plantarum* ATCC 8014 as the outgroup in the phylogenetic tree of the *Lactobacillus* genus and *Lactobacillus jensenii* FDAARGOS 749 as the outgroup for *Lactobacillus delbrueckii.*

We used the amino acid sequences of each protein in the PrtB peptidase analysis. A BLAST search against the non-redundant protein database of the NCBI was done to identify the sequences that have the greatest similarity to the *L. delbrueckii* spp *bulgaricus* 2038 PrtB. We selected sequences from the orthologous group (COGs) in the pangenome analysis for beta-galactosidase analyses. For both proteins, we used MUSCLE v3.8.31 (Edgar 2004) for MSA and IQ-TREE v2.0.6 (Nguyen et al. 2015), for tree calculation. ITol v6 (Letunic and Bork 2021) was used for the visualization and editing of all trees.

### Pangenomic analysis

We analyzed the composition of the pangenome using the PEPPAN pipeline (Zhou et al. 2020) as it gives the best results when analyzing at the genus level. To have a coherent annotation system, all downloaded genomes were re-annotated using the PROKKA program (Seemann 2014) under default conditions. Subsequently, we used PEPPAN with the following conditions:—match_identity 0.4—clust_identity 0.5—pseudogene 0.9—min_cds 300 using the 1020 proteomes as defined previously. The most relevant cut-off is the minimum identities of mmseqs clusters (clust_identity), as higher values give a very large number of false singletons. The resulting data were processed using PEPPAN_parser under the following conditions: -t-c-a 95. The resulting presence and absence file, covering all genes in the pangenome and all genomes, were further processed using Scoary (Brynildsrud et al. 2016) under the conditions: -p 1E-5—c BH—no_pairwise -s 2 and a traits file describing the isolation origin of each strain. The results were analysed to identify which genes are strongly associated with particular traits.

### Analysis of specific genes for the utilization of specific carbohydrates

We searched for key enzymes in the degradation pathways of galactose (galactose mutarotase), fructose (phosphofructokinase), mannose (class II mannose-6-phosphate isomerase), starch (alpha-amylase), lactose (beta-galactosidase) and rhamnose (l-rhamnose isomerase).

Similarly, for amino acid biosynthesis, we searched for key enzymes in the synthesis of amino acids lysine (diaminopimelate decarboxylase), arginine (argininosuccinate lyase), cysteine (serine* O*-acetyltransferase and cysteine synthase) threonine (threonine synthase), proline (ornithine cyclodeaminase and glutamate-5-semialdehyde dehydrogenase), asparagine (aspartate—ammonia ligase), methionine (methionine synthase), and for aromatic amino acids tryptophan, tyrosine and phenylalanine (shikimate kinase, shikimate dehydrogenase and tryptophan synthase).

We use GeneSpy (Garcia et al. 2019) for the identification of the genomic context, using the assembly statistic report and the gff files from the NCBI database.

### CAZymes

The CAZyme modules were annotated using dbCAN2 (Zhang et al. 2018); (hmmscan cut-offs: E-value < 1e-15, coverage > 0.35, DIAMOND cutoffs: E-value < 1e-102, Hotpep (Frequency > 2.6, Hits > 6). The integrated matrices were written using R, bash, Perl, and Python. Statistical analyses were performed using R-v. 4.2.3 (R Core Team 2023). Using the Bray–Curtis dissimilarity index to calculate distance matrices relative to the taxa abundance group at the class level of taxonomy and CAZyme composition, a principal coordinate analysis (PCoA) was performed. PCoAs were visualized using the vegan, pragma (Oksanen 2010), and geosphere (Hijmans, RF 2020) R packages.

## Results

### Habitat adaptation drives evolutionary diversity

The reclassification of the *Lactobacillus* genus has unveiled significant genomic diversity within lactobacilli (Zheng et al. 2020). This discovery raises questions about how the genus has evolved and diversified across different habitats. Lactobacilli are reported to inhabit four primary environments: vertebrates, insects, free-living settings, and nomadic niches (Duar et al. 2017). Given this, we investigated whether adaptation to these specific habitats correlates with phylogenetic diversification. To assess strain diversification, we constructed a distance tree using 1020 complete *Lactobacillus* genomes available in the NCBI database (Fig. S1), including strains classified as uncultured and those without a named species (designated as ‘sp.’).

It is important to note the sampling bias in genome availability across species. *Lactobacillus crispatus* has the highest number of reported genomes, which likely contributes to its distinct clustering in a separate clade within the genus, characterized by low genetic distance among its strains. In contrast, *Lactobacillus iners* and *Lactobacillus jensenii* exhibit high genetic diversity, as evidenced by their extensive branching and close genetic distances. Notably, these three species—*L. crispatus*, *L. iners*, and *L. jensenii*—are strongly associated with the vaginal microbiota (Chee et al. 2020; France et al. 2016; Petrova et al. 2017). However, *L. iners* is exclusively linked to the vaginal microbiota.

**Fig. 1:**
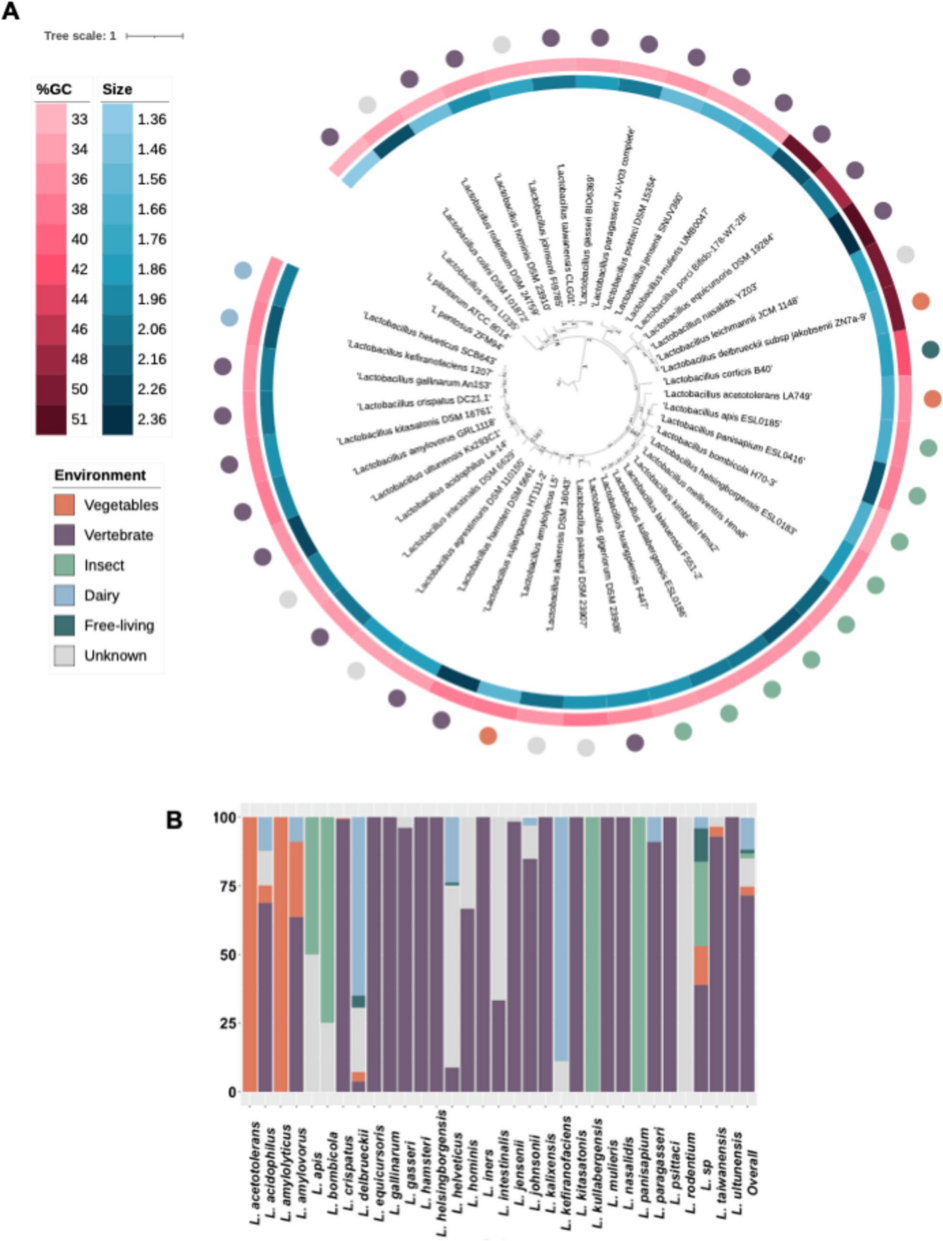
**a** Maximum likelihood phylogenetic tree of the 43 reference genomes of the *Lactobacillus* genus. *L. plantarum* ATCC 8014 and *L. pentosus* ZFM94 were used as outgroup. The size and the %GC are represented from the inner to the outer circle. **b** Bar chart of the percentage of habitats in species of the *genus Lactobacillus.* We used ITol v5 for the visualization and modification of the tree (Letunic and Bork 2021b)

Additionally, strains classified as uncultured form a distinct group with the longest branches in the distance tree. These long branches may result from the identification of these genomes through metagenomic analyses, potentially leading to misclassification. The distance matrix-based tree is useful for screening a large number of genomes and highlighting differences between the compared sequences. However, it does not allow for the establishment of phylogenetic relationships. To address this, we constructed a Maximum Likelihood (ML) phylogenetic tree using PhyloPhlAn (Asnicar et al. 2020) (Fig. 1a). This tree included 45 species with reference genomes available in the NCBI database (Table S1). The resulting phylogeny reveals a pronounced split, dividing the genus into two well-supported clades with 100% bootstrap support.

The first clade comprises *L. iners*, the most divergent species, along with *L. colini*, *L. rodentium*, *L. hominis*, *L. johnsonii*, *L. taiwanensis*, *L. gasseri*, and *L. paragasseri*. The second clade encompasses the remaining species, exhibiting multiple speciation events. Upon analyzing GC content and genome size across the genus, we observed no significant differences in GC content between the two clades overall. Notably, the *Lactobacillus delbrueckii* clade exhibits a GC content of approximately 50%, markedly higher than the genus average of 30%. In terms of genome size, no substantial differences were found between the clades, except for *L. iners* in the first clade, which has a significantly smaller genome size (average of 1.3 Mb).

The discrepancies observed in the distance tree and the ML phylogenetic tree indicate that certain genomic characteristics are not solely dictated by phylogenetic relationships. These variations may be attributed to habitat adaptation, as has been previously reported in other bacterial genera (Boscaro et al. 2017; Rocha and Danchin 2002).

The habitat distribution of *Lactobacillus* isolates was analyzed based on their isolation sources. It is important to acknowledge a significant bias in the sampling habitats. The number of reported genomes for each species may vary depending on research interest, leading to an overrepresentation of certain habitats. For example, the majority of *Lactobacillus* strains have been isolated from vertebrates (including humans and animals), followed by dairy products (Fig. 1b). In contrast, strains from vegetables and free-living environments are less represented. This distribution should be interpreted with caution, as it does not necessarily indicate that these strains are incapable of inhabiting other environments.

A detailed analysis of habitat distribution within the genus revealed that only three species have strains isolated from free-living environments: *L. delbrueckii*, *L. acetotolerans*, and *L. helveticus* (Fig. 1b). In contrast, certain species have been exclusively isolated from insect-associated habitats, including *L. apis*, *L. bombicola*, *L. helsingborgensis*, *L. kullabergensis*, and *L. panisapium*. Furthermore, some species demonstrate the ability to inhabit a broad range of environments. For example, strains of *L. crispatus* have been isolated from vertebrate-associated habitats, as well as from dairy products and vegetables, highlighting their environmental versatility. This distribution may result from genetic drift; however, it is more likely the outcome of adaptation through adjustments in genomic content, including both gene acquisition and loss.

### Habitat-specific genes

#### Pangenome analysis

Our previous analysis revealed that *Lactobacillus* species have adapted to a wide range of environments. A species capable of surviving in a specific habitat typically possesses traits that facilitate nutrient uptake, enable survival under environmental conditions, and allow competition with other microorganisms. To identify the specific traits that enable *Lactobacillus* species to thrive in diverse habitats, we conducted a pangenomic analysis using 1020 complete genomes from the *Lactobacillus* genus (Table S2).

The *Lactobacillus* pangenome comprises a total of 25,712 genes, categorized as follows: 0 core genes (present in 99–100% of genomes), 209 softcore genes (present in 95–99% of genomes), 2120 shell genes (present in 15–95% of genomes), and 23,383 cloud genes (present in 0–15% of genomes). PEPPAN (Zhou et al. 2020) employs a strict cutoff for core genome classification, which explains the absence of core genes in our analysis. However, the 209 softcore genes are primarily associated with central metabolism and essential cellular functions, which are generally conserved across all *Lactobacillus* species. Interestingly, 91% of the genes in the pangenome are classified as cloud genes.

To identify correlations between gene presence and specific habitats, we utilized Scoary (Brynildsrud et al. 2016), a software tool designed to determine statistical associations between phenotypes and genotypes. In this analysis, the habitat from which each strain was isolated was treated as the phenotype. After identifying the genes, we applied a 90% specificity cutoff to select those positively correlated with each habitat and classified them according to their COG (Clusters of Orthologous Groups) categories (Table S3). The sequences identified are available on GitHub (https://github.com/RafaelLopez-Sanchez/Lactobacillus_genomics).

**Fig. 2:**
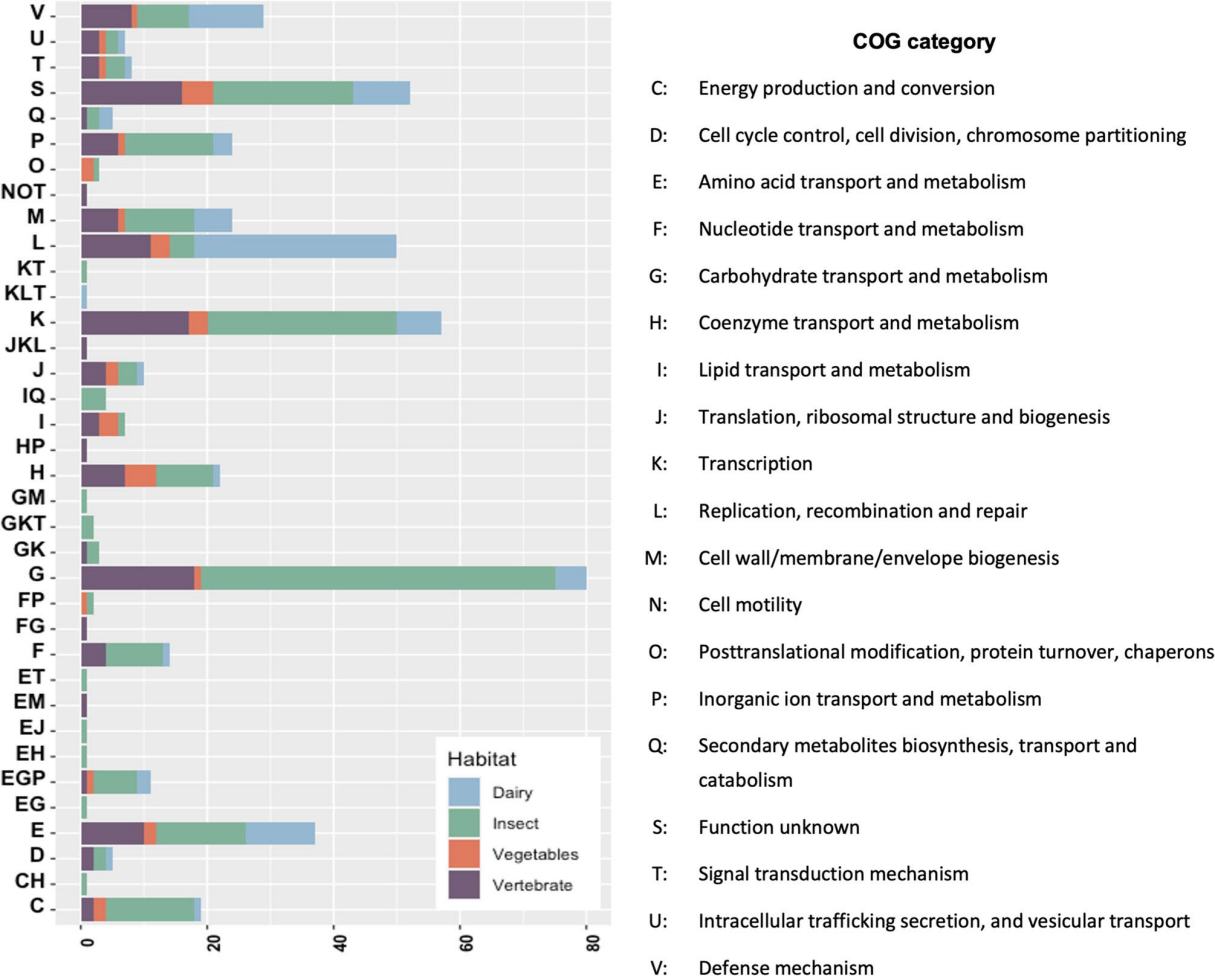
COG category of genes with a positive statistical correlation between phenotype and genotype determined by Scoary with a cutoff of 95% specificity

No genes exhibited a correlation greater than 95% with the free-living habitat. Among the other habitats, insect-associated environments showed the highest number of specific genes. The genes most strongly correlated with insect-associated habitats were involved in carbohydrate transport and metabolism, followed by those related to transcription, energy production, and amino acid transport and metabolism. For vertebrate-associated habitats, highly correlated genes were also primarily involved in carbohydrate transport and metabolism, as well as in transcription, replication, recombination, and repair. In dairy-associated environments, the predominant correlated functions were replication and recombination repair. No functions were found to be over-represented in the vegetable-associated habitat; however, genes related to coenzyme transport and metabolism, replication and recombination repair, and lipid transport and metabolism were the main functions represented in this environment (Fig. 2). Overall, carbohydrate transport and metabolism, along with amino acid transport and metabolism, were the primary functions strongly correlated with the various habitats. An interesting observation is that the number of hypothetical genes having a clear association to specific traits is around 50% for all genes with a higher than 90% specificity in the Scoary analysis (Table S3). These are clearly pertinent candidates for further research as some have a 100% specificity score.

#### Adaptation to nutrients

Nutrient uptake and metabolism are the primary strategies altered during habitat adaptation. The two key functions most strongly correlated with different habitats are carbohydrate and amino acid transport and metabolism, both directly linked to the availability of nutrients in their respective environments (Yu et al. 2009). Consequently, we analyzed specific genes associated with these functions, as well as carbohydrate-active enzymes (CAZymes), within the *Lactobacillus* genomes. We chose to focus on key enzymes within metabolic pathways rather than on transport mechanisms. This approach was based on the observation that many transporters lack substrate specificity, making it difficult to identify subtle differences in habitat adaptation among different species.

### Specific genes of carbohydrate metabolism

**Fig. 3:**
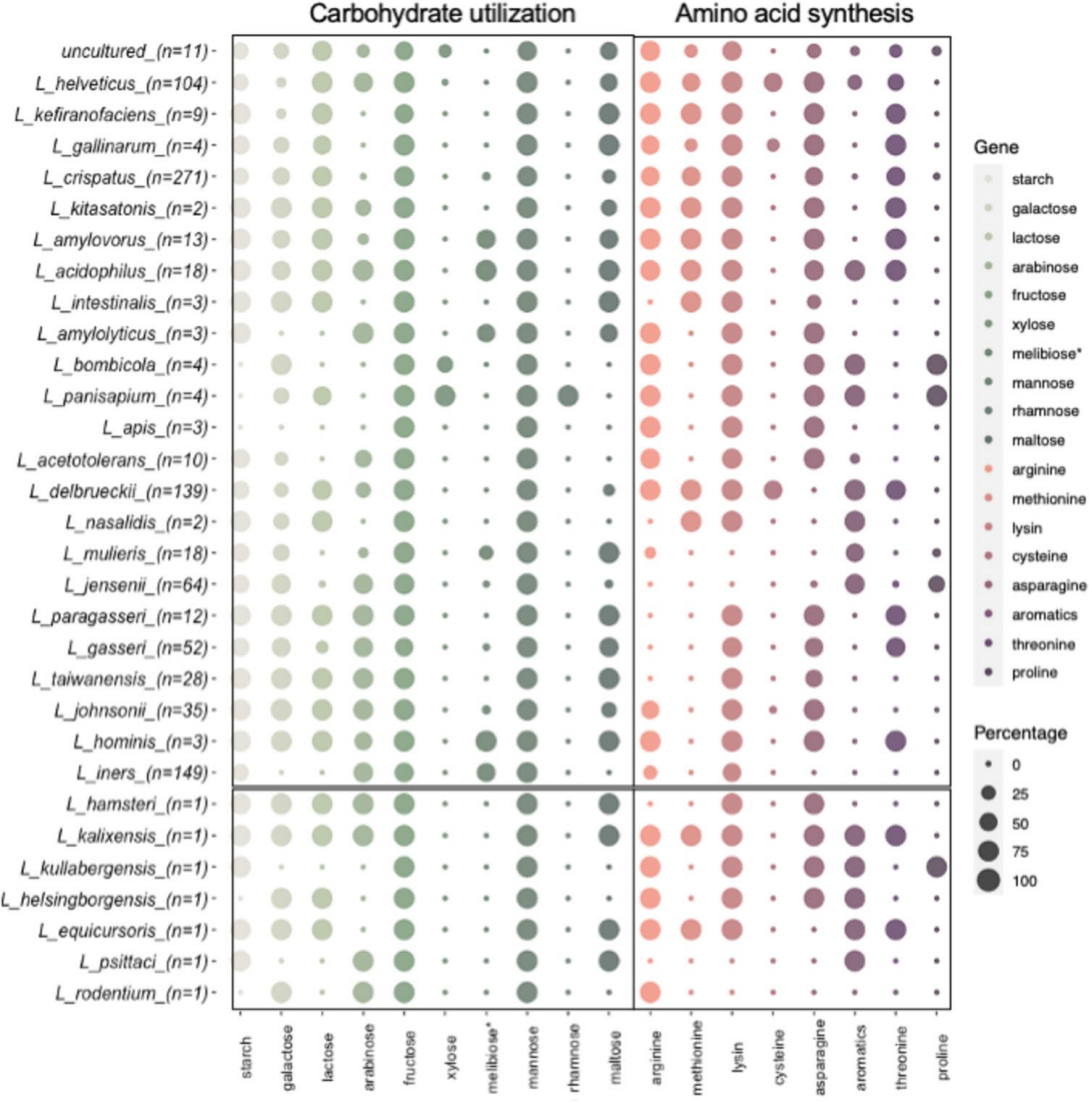
Bubble plot comparing key enzymes of carbohydrate metabolic utilization genes and amino acid biosynthetic pathways between species. The size of the bubble indicates the percentage presence of each enzyme within each species

Certain sugars are exclusive to specific environments, while others, such as glucose, are widely distributed. Considering this, we focused on carbon sources that may drive habitat adaptation. The metabolism of mannose and fructose is common across all *Lactobacillus* species. In contrast, rhamnose metabolism is restricted to *Lactobacillus panisapium*, and only *L. panisapium* and *Lactobacillus bombicola* can metabolize xylose; both species are exclusively isolated from insect guts (Fig. 3). Interestingly, the ability to degrade starch and maltose is ubiquitous among all species except *L. helsingborgensis*, *L. bombicola*, *L. panisapium*, and *L. apis*, all of which are associated with insects. Additionally, melibiose utilization is observed in *L. iners*, *L. hominis*, *L. mulieris*, *L. amylolyticus*, *L. acidophilus*, *L. ultunensis*, and *L. amylovorus*. These species are primarily associated with vertebrates, with the notable exception of *L. amylolyticus*, which has been isolated from vegetable habitats.

Arabinose utilization was detected in nearly all *Lactobacillus* species, except for those primarily associated with insects (*L. apis*, *L. panisapium*, *L. bombicola*, *L. helsingborgensis*), vertebrates (*L. nasalidis*, *L. equicursoris*, *L. kullabergensis*, *L. intestinalis*, *L. crispatus*, *L. gallinarum*), and dairy environments (*L. kefiranofaciens*) (Fig. 3). Similarly, galactose and fructose metabolism are prevalent across most *Lactobacillus* species, with the exceptions of *L. apis* and *L. kullabergensis* (insect-associated), *L. iners* (exclusively vaginal microbiota), *L. amylolyticus* (vegetable-associated), and *L. psittaci* (vertebrate-associated) (Fig. 3). This distribution highlights the specialized metabolic capabilities of certain *Lactobacillus* species with their respective habitats.

As was seen in the pangenome analysis, the species associated with insects are those with a higher number of genes with a strong correlation with the environment.

### Specific genes of amino acid synthesis

The correlation between habitat adaptation and amino acid transport and metabolism highlights a crucial aspect of bacterial evolution. It has been observed that bacteria thriving in nutrient-rich environments often lose the ability to synthesize metabolites that are readily available in their surroundings (Yu et al. 2009). In lactobacilli, strains with smaller genomes have been reported to exhibit losses in amino acid, lipid, and nucleotide metabolism pathways (Ardalani et al. 2024). Lactobacilli strains isolated from environments such as dairy products, fermented vegetables, and animal guts show a higher prevalence of auxotrophies. The presence of these auxotrophies, particularly those related to amino acid biosynthesis, indicates nuanced adaptation to specific environments.

To further investigate these adaptations, we examined key enzymes involved in biosynthetic pathways. The proline and cysteine biosynthesis pathways were found to be incomplete in several species. Conversely, lysine synthesis was prevalent in most species, except for *L. mulieris*, *L. jensenii*, and *L. psittaci*. These species belong to the same phylogenetic branch and are associated with vertebrates. Additionally, the methionine biosynthesis pathway was incomplete in species associated with both insects and vertebrates (Fig. 3).

These analyses suggest that carbohydrate metabolism and amino acid synthesis functions are closely linked to the environmental niches of bacterial species. Additionally, closely related species exhibit shared phylogenetic characteristics. This raises an important question: Do these species inherently possess genomic traits that enable their proliferation in similar environments, or have evolutionary pressures shaped them into comparable forms over time? It is plausible that the convergence of genomic characteristics, driven by common environmental pressures, has led to their phylogenetic clustering.

### CAZyme composition

**Fig. 4:**
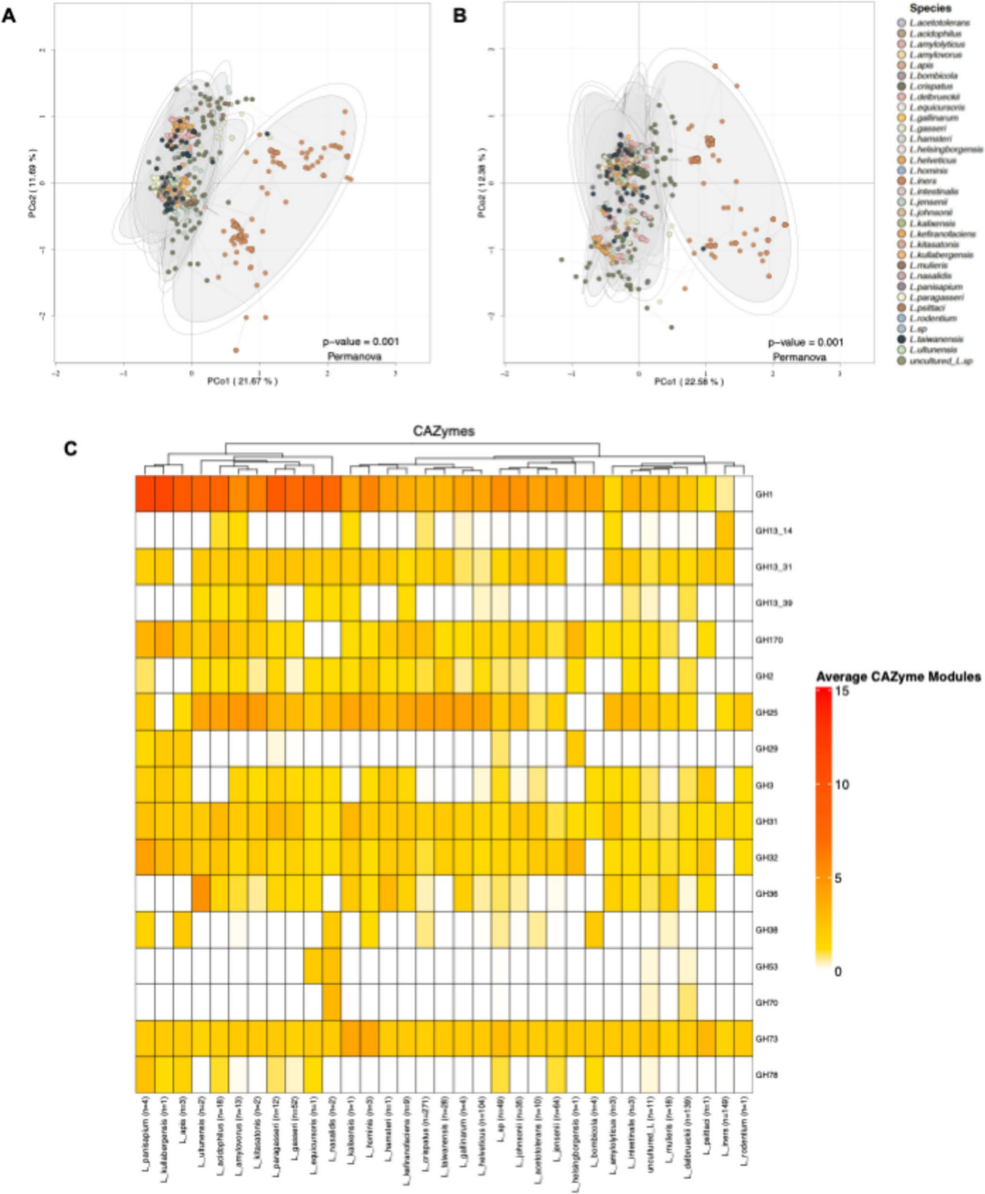
Principal Coordinate Analysis (PCoA) performed on *Lactobacillus* genomes to construct a Bray–Curtis dissimilarity matrix. The occurrence of taxonomic species in each genome is color-coded. For visualization purposes, data were transformed to the cubic root, based on **a** total CAZyme module counts and **b** total Glycoside Hydrolases (GHs). **c** Heat map of the most abundant carbohydrate-activated enzyme (CAZymes) modules found in *Lactobacillus* species on average per species. In parentheses, the number of genomes from each species is indicated

To investigate the relationship between carbohydrate metabolism and habitat adaptation or phylogenetic distributions, we conducted a principal coordinate analysis (PCoA) based on the CAZyme composition of *Lactobacillus* strains using the Bray–Curtis dissimilarity matrix. Notably, this analysis did not reveal a clear separation among the various environments of the *Lactobacillus* genomes, accounting for 33.22% of the variance in CoA1 and CoA2 (Fig. S2). However, when we performed the same analysis at the species level, based on CAZyme composition and using the Bray–Curtis dissimilarity matrix, a distinct separation emerged exclusively between *L. iners* genomes and the other species, also explaining 33.22% of the variance in CoA1 and CoA2. This separation was evident for both all CAZymes and specifically for Glycosyl Hydrolases (GHs) (Fig. 4a and b).

Further analysis focused exclusively on glycosyl hydrolase enzymes due to their direct role in carbohydrate utilization. Specifically, the GH1 family was prominently represented in the first clade, which is further divided into two subclades (Fig. 4c). The first subclade includes species associated with insects, such as *L. panisapium*, *L. kullabergensis*, and *L. apis*, along with species related to the gastrointestinal tracts of vertebrates. In contrast, the second subclade predominantly comprises vertebrate-associated species, including *L. equicursoris*, *L. gasseri*, *L. paragasseri*, *L. kitasatonis*, *L. amylovorus*, *L. acidophilus*, and *L. ultunensis*. The GH1 family includes a diverse range of functions, with β-glucosidases and β-galactosidases being the most common enzymes. Their substrates encompass polyphenols, milk polysaccharides, pectin, xylan, arabinan, peptidoglycan, host glycans, and beta-glucans, among others.

The GH31 and GH73 families are ubiquitous among all *Lactobacillus* species analyzed. Within the GH31 family, enzymes such as α-glucosidases, α-xylosidases, isomaltosyltransferases, maltases/glucoseamylases, and sulfoquinovosidases have been identified, with starch, glycan, and sucrose serving as their primary substrates. The GH73 family predominantly comprises enzymes that cleave the β-1,4-glycosidic linkage between the N-acetylglucosaminyl (NAG) and* N*-acetylmuramyl (NAM) moieties in bacterial peptidoglycans. Notably, the GH29 family is exclusive to insect-associated species, including *L. panisapium*, *L. apis*, *L. kullabergensis*, and, within a distinct clade, *L. helsingborgensis*. Enzymes within the GH29 family function primarily as exo-acting α-fucosidases, with no other enzymatic activities observed. Although fucose is present in the* N*-glycan structures of mammals, plants, and insects, variations in* N*-glycan structures among these groups play a pivotal role in host-microbial interactions. The exclusive presence of the GH29 family in insect-associated species suggests a specialized mechanism for colonization or interaction with the host within these environments.

Among the genes analyzed, those associated with insect habitats are the most distinctive. This divergence is likely due to the unique dietary and gastrointestinal environments of insects. In contrast, vertebrate-associated species exhibit a more generalized distribution of functions, with fewer genes directly linked to habitat adaptation. This suggests that the diverse and varied diets of vertebrates facilitate the acquisition of genes that enable *Lactobacillus* species to survive and integrate into the vertebrate microbiota.

Although the principal coordinate analysis did not reveal clear separations based on environmental habitats, a distinct clustering was observed between *L. iners* and the other species. The prominence of specific glycosyl hydrolase enzyme families, particularly in insect-associated species, underscores the influence of dietary and gastrointestinal factors on bacterial genomic composition. Furthermore, the broader distribution of functions among vertebrate-associated species suggests a more generalized adaptation to diverse nutritional and environmental conditions. The exceptional case of *L. iners*, with its specialized enzymatic profile in the vaginal microbiota, highlights the importance of niche-specific adaptations in bacterial evolution.

### Adaptation to artificial environments: specific example of dairy

Dairy is one of the primary environments from which *Lactobacillus* strains have been isolated (Fig. 1b). This environment is particularly significant as it represents one of the most widely utilized artificial habitats globally. Consequently, we analyzed the distribution of two key functions essential for growth in dairy environments: lactose and casein hydrolysis. The enzyme responsible for the accelerated growth of *Lactobacillus delbrueckii* in milk is the serine protease PrtB, which is involved in casein hydrolysis (Li et al. 2019). We searched for PrtB within the *Lactobacillus* pangenome, where it is annotated as peptidase S8. PrtB was more frequently found in *L. delbrueckii* and some strains of *L. helveticus*, *L. johnsonii*, *L. kefiranofaciens*, and *L. amylovorus* genomes.

**Fig. 5:**
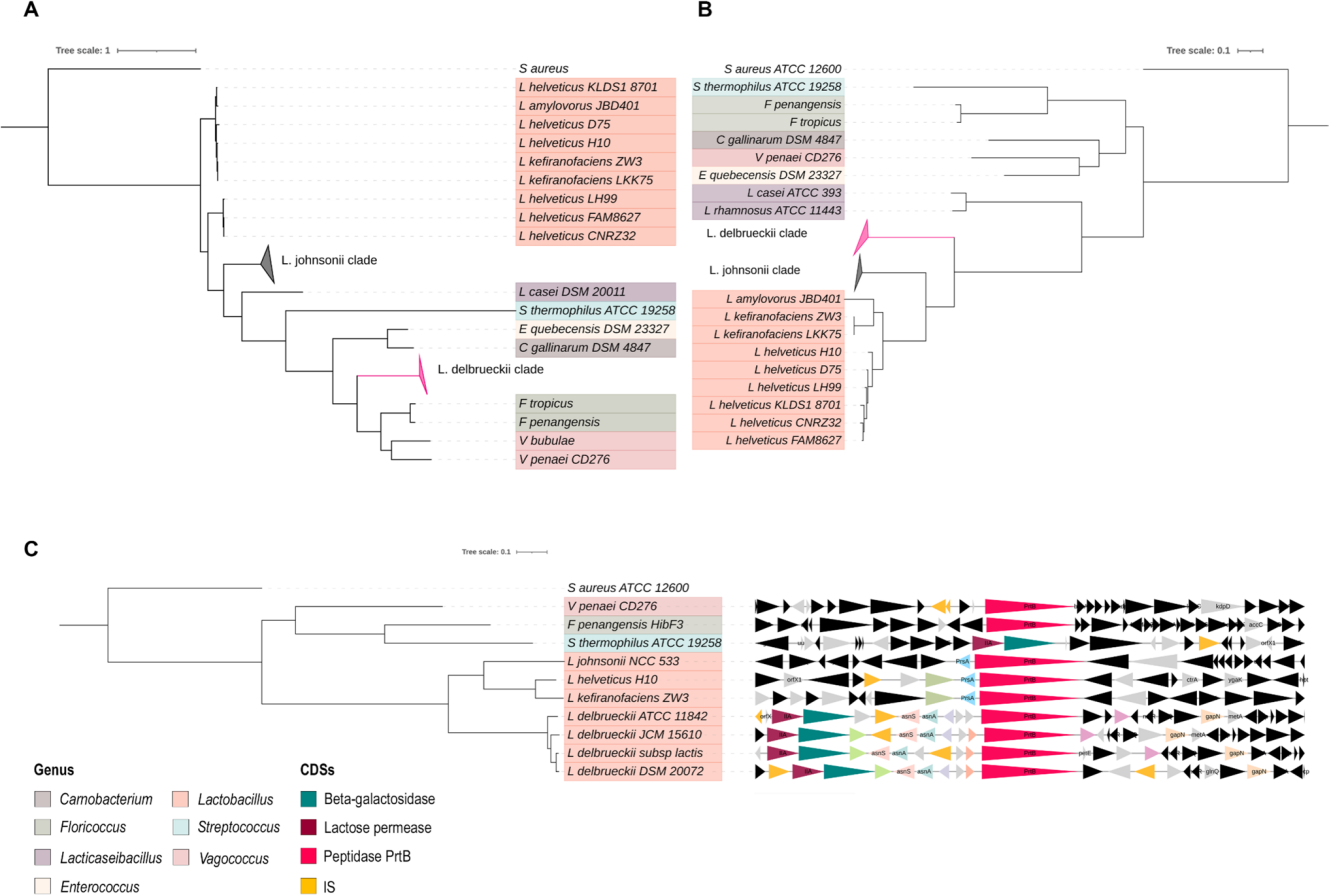
**a** Comparative phylogeny between the PrtB-based Maximum Likelihood tree (left) and the complete genome-based maximum likelihood tree of *L. delbueckii* species (right). We use *S. aureus* ATCC 12600 as an outgroup. Visualization and tree modification were performed with ITol v5 (Letunic and Bork 2021b). The color in the tips indicates the different genus. **b** Maximum likelihood phylogenetic tree of *L. delbueckii* in the context of key species of the genus *Lactobacillus*, *Streptococcus, Floricoccus,* and *Vagococcus* (left), compared with the genomic context of proteinase PrtB (right). The genomic context was made with GeneSpy software (Garcia et al. 2019)

Using the PrtB sequence from *L. delbrueckii* ssp. *bulgaricus* 2038 (industrial strain), we conducted a BLAST search against the NCBI database to identify related proteins. Surprisingly, PrtB showed high similarity to peptidase S8 from *Floricoccus penangensis*, *Floricoccus tropicus*, *Vagococcus penaei*, *Carnobacterium gallinarum*, and *Enterococcus quebecensis*. A Maximum Likelihood (ML) phylogenetic tree constructed using these proteins revealed that *L. delbrueckii* PrtB clusters with sequences from *Floricoccus* and *Vagococcus* species (Fig. 5a), which are primarily associated with plants and animals (Chuah et al. 2017; Tak et al. 2017; Wu et al. 2020). The prtB gene is located in a genomic region containing multiple insertion sequences (IS) and transposases (Fig. 5c), suggesting that PrtB was acquired through horizontal gene transfer (HGT), potentially from plant-associated species. Although phages have been associated with horizontal gene transfer (HGT) events in lactobacilli (Pei et al. 2021), the analysis of surrounding genome sequences reveals no phage genes within 100 kb, nor any evidence of potential vectors involved in HGT.

Additionally, genes encoding beta-galactosidase and lactose permease are located near the prtB gene in *L. delbrueckii* genomes (Fig. 5c). Therefore, we searched for beta-galactosidase genes within the *Lactobacillus* pangenome. Beta-galactosidase is a key indicator of strain adaptation to habitats used for producing fermented dairy products and to species associated with mammals (Honda et al. 2007; Kittibunchakul et al. 2019). Although no beta-galactosidase genes were annotated in *L. delbrueckii* genomes, BLAST searches using the beta-galactosidase sequence from *Streptococcus thermophilus* identified sequences annotated as domains of unknown function (DUF4981). Based on this finding, we propose that the ability to metabolize lactose was acquired by HGT from *S. thermophilus*. However, further analysis is necessary to elucidate the origin of this enzyme.

## Discussion

In recent years, due to the development and improvement of sequencing technologies, there has been an increase in the number of LAB isolated from diverse sources. The increase in the number of species was accompanied by an increase in the diversity of this group. For this reason, a reclassification of lactobacilli was proposed that allows better separation of species according to a polyphasic approach (Zheng et al. 2020). However, despite this new reclassification, there is still a great variation within the *Lactobacillus* genus, probably related to the environments in which they can be found. The phylogenetic analysis of *Lactobacillus* strains reveals different clustering patterns, suggesting their adaptation to specific habitats. In particular, the *L. delbrueckii* clade exhibits a higher GC content than other species, which may indicate unique evolutionary pressures. The difference in GC% between the species located in the phylogenetic clade of *L. delbrueckii* and the rest of the genus is around 20%, five times more than that observed for other genera of bacteria. On average, a very similar genome size (2 Mb) is observed, except for *L. iners* (1.4 Mb), *L. jensenii* and *L. mulieris* (1.6 Mb), and *L. acetotolerans* (1.5 Mb). The differences between *L. delbrueckii* and the rest of the species have been proposed to be due to its rapid and specialized adaptation to artificial environments such as yogurt (van de Guchte et al. 2006). However, in recent years different subspecies have been isolated from plant sources (Michaylova et al. 2007), such as *L. delbrueckii* ssp *sunkii* (Kudo et al. 2012), which also have a high GC content. Another interesting example is *L. iners*, which consistently clustered separately from the rest of the genus in all our analyses. This separation was also observed in a genome-scale metabolic model, where *L. iners* diverged from other members of the genus, suggesting a unique metabolic profile (Ardalani et al. 2024). This separation may be related to the specialization of this species in the vaginal environment.

On the other hand, the adaptation of lactobacilli to different environments, such as fermented foods, has been reported to be mediated by the acquisition and loss of functions (Makarova and Koonin 2007). Most *Lactobacillus* strains have been isolated from vertebrates and dairy products, with fewer examples found in vegetables and free-living environments. Interestingly, only three species have been reported in free-living habitats, a fact that may be influenced by limited sampling. Despite this, an intriguing study from Bulgaria demonstrated that *L. delbrueckii* strains, typically linked to dairy and vertebrate environments, were isolated from plants in a free-living context (Michaylova et al. 2007), highlighting the need for a broader environmental sampling.

The genus *Lactobacillus* exhibits a high degree of genomic complexity, underscoring its ability to adapt to various environments. This diversity suggests that various adaptive strategies can coexist within the same environment. Analyses of carbohydrate-active enzymes (CAZy), amino acid biosynthetic pathways, and carbohydrate metabolism genes illustrate significant metabolic differences among species. However, no clear relationship was found between habitat adaptation and phylogenetic closeness, as strain-specific traits dominate, and universal markers for habitat adaptation are rare. In insect-associated habitats, the highest number of unique genes were found, predominantly related to carbohydrate use and amino acid biosynthesis.

Furthermore, analysis of the *Lactobacillus* pangenome, using specific cut-off parameters, revealed that there are no core genome genes. However, genes involved in central metabolism and common to the genus are part of the soft-core genome. This could be caused by the fragmentation in contigs of the genomes in the database.

The study of horizontal gene transfer (HGT) suggests that PrtB protease, important for the adaptation of *L. delbrueckii* to dairy environments through its role in casein hydrolysis, was likely acquired from plant-associated species. This highlights how a crucial trait for adaptation to dairy environments may have been obtained through HGT. Lastly, the analysis identified numerous genes with strong statistical correlations to specific habitats, though many are hypothetical proteins with unknown functions. This strong correlation indicates an important function for these proteins in the adaptation to these environments. Future research will be necessary to elucidate their roles, which could be key to understanding the specific mechanisms that allow *Lactobacillus* strains to thrive in diverse environments. This analysis also opens new avenues of research through the identification of genes involved in the colonization and adaptation to specific habitats. Their roles and in particular that of unannotated or hypothetical genes need to be addressed. Overall, the study highlights the complex interplay between genomic traits, environmental pressures, and habitat-specific adaptations in shaping the evolutionary trajectory of *Lactobacillus* species.

## Supplementary Information

Below is the link to the electronic supplementary material.

Supplementary file1 (TXT 69 KB)

Figure S1. Neighbour-joining distance tree of the 1020 complete genomes of the* Lactobacillus* genus. The colour strip shows the species. We used ITol v5 for the visualisation and modification of the tree (Letunic and Bork 2021b).
Supplementary file2 (TIFF 24 MB)

Figure S2. Principal Coordinate Analysis (PCoA) identified within* Lactobacillus* genomes to construct a Bray-Curtis dissimilatory matrix. The occurrence of habitat and taxonomy of each genome is colour coded. For visualization purposes, data were transformed to the cubic root, based on A) total CAZyme module counts and B) total Glycoside Hydrolases (GHs).
Supplementary file3 (TIFF 1.99 MB)

Figure S3. Heat map of the most abundant carbohydrate-activated enzyme (CAZymes) modules found in* Lactobacillus* species on average per species. In parentheses, the number of genomes from each species is indicated.
Supplementary file4 (TIFF 24 MB)

Supplementary file5 (XLSX 12 KB)

Supplementary file6 (XLSX 24 KB)

Supplementary file7 (XLSX 611 KB)

## Data Availability

Data is provided within the manuscript or supplementary information files and the raw results of our analyses are available at: https://github.com/RafaelLopez-Sanchez/Lactobacillus_genomics .
