# Supplementary figures and images for: Genomic insights into habitat adaptation of Lactobacillus species
Source: World J Microbiol Biotechnol. 2025 Feb 4;41(2):61. doi: 10.1007/s11274-025-04275-0 (PMC11790720; doi:10.1007/s11274-025-04275-0)

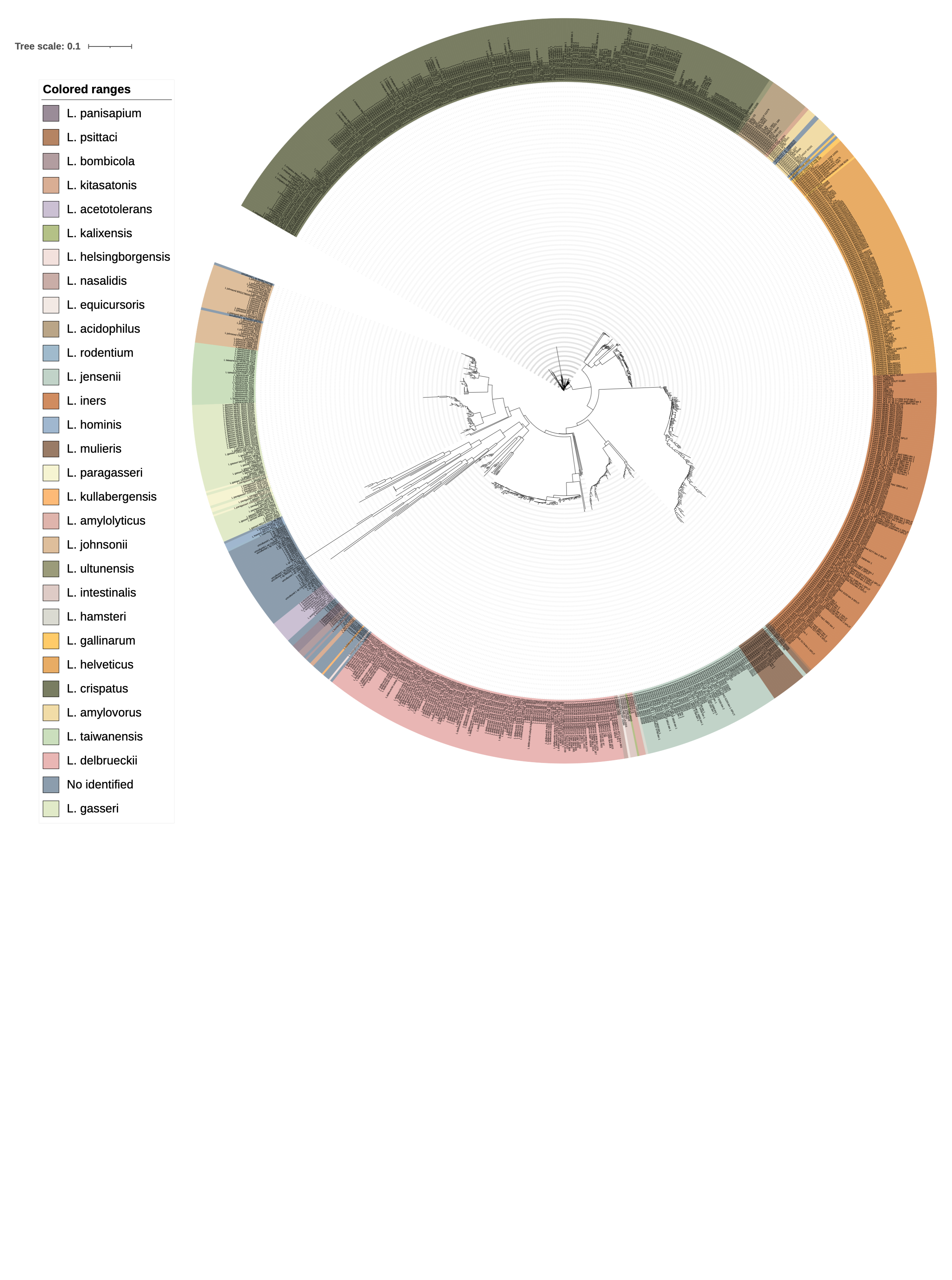

Supplement: Supplementary file 2 — Figure S1. Neighbour-joining distance tree of the 1020 complete genomes of the Lactobacillus genus. The colour strip shows the species. We used ITol v5 for the visualisation and modification of the tree (Letunic and Bork 2021b). Supplementary file2 (TIFF 24 MB) [file 11274_2025_4275_MOESM2_ESM.tiff]

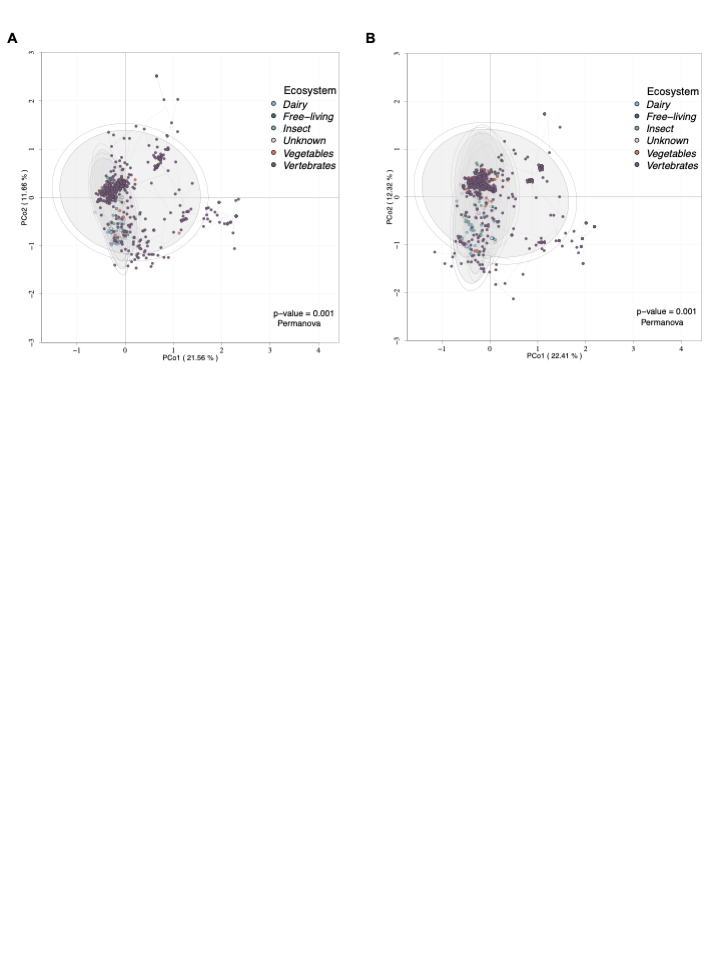

Supplement: Supplementary file 3 — Figure S2. Principal Coordinate Analysis (PCoA) identified within Lactobacillus genomes to construct a Bray-Curtis dissimilatory matrix. The occurrence of habitat and taxonomy of each genome is colour coded. For visualization purposes, data were transformed to the cubic root, based on A) total CAZyme module counts and B) total Glycoside Hydrolases (GHs). Supplementary file3 (TIFF 1.99 MB) [file 11274_2025_4275_MOESM3_ESM.tiff]

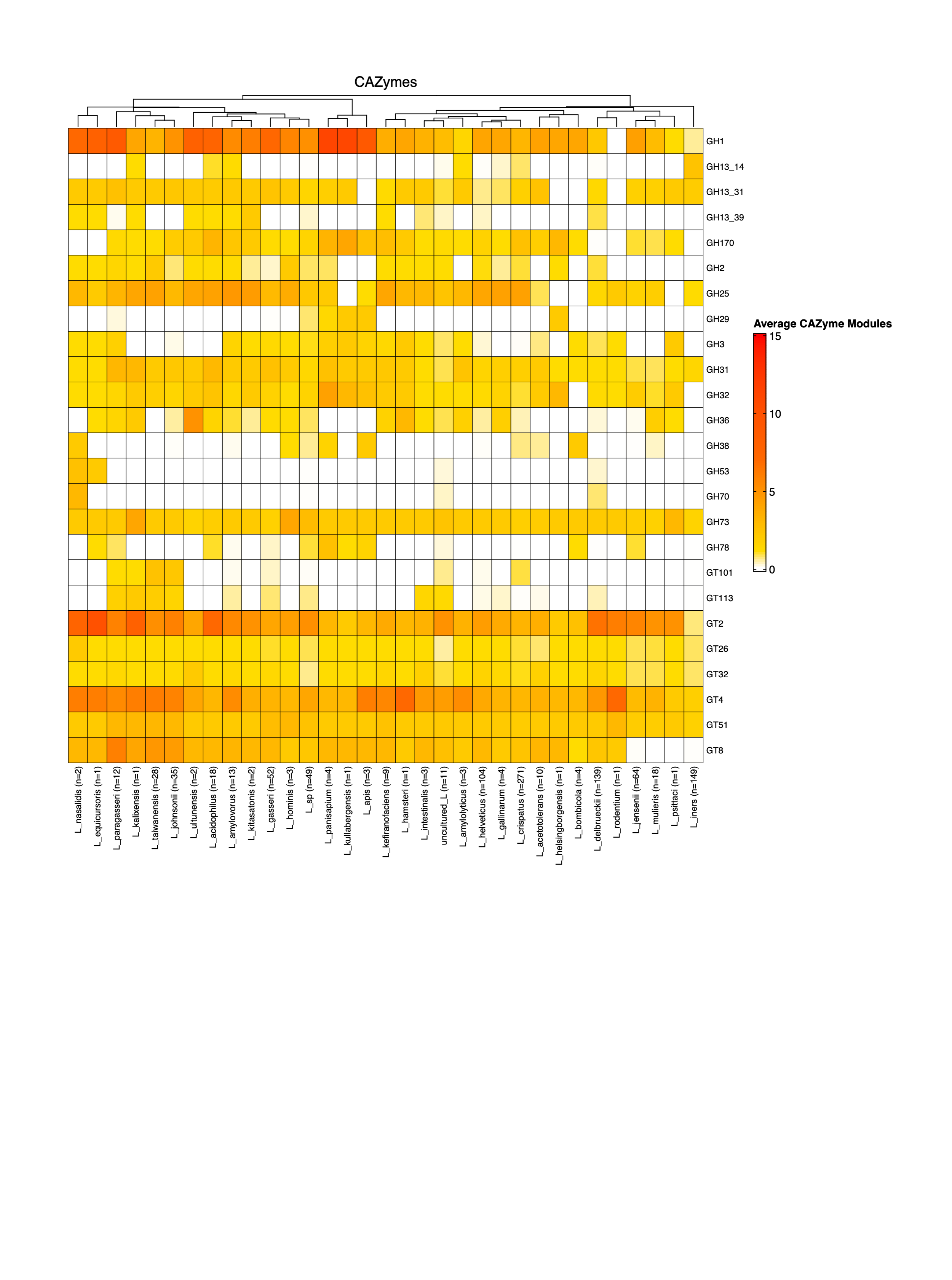

Supplement: Supplementary file 4 — Figure S3. Heat map of the most abundant carbohydrate-activated enzyme (CAZymes) modules found in Lactobacillus species on average per species. In parentheses, the number of genomes from each species is indicated. Supplementary file4 (TIFF 24 MB) [file 11274_2025_4275_MOESM4_ESM.tiff]
